# Blau syndrome with hypertension and hepatic granulomas: a case report and literature review

**DOI:** 10.3389/fped.2023.1063222

**Published:** 2023-07-27

**Authors:** Fangling Yao, Bei Tan, Di Wu, Min Shen

**Affiliations:** ^1^ Department of Rheumatology and Clinical Immunology, Chinese Academy of Medical Sciences & Peking Union Medical College, National Clinical Research Center for Dermatologic and Immunologic Diseases (NCRC-DID), Ministry of Science & Technology, State Key Laboratory of Complex Severe and Rare Diseases, Peking Union Medical College Hospital (PUMCH), Key Laboratory of Rheumatology and Clinical Immunology, Ministry of Education, Beijing, China; ^2^ Department of Rheumatology and Immunology, Zhuzhou Central Hospital, Zhuzhou, China; ^3^ Department of Gastroenterology, Peking Union Medical College Hospital, Chinese Academy of Medical Science & Peking Union Medical College, Beijing, China

**Keywords:** Blau syndrome, hypertension, hepatic granuloma, hepatosplenomegaly, digestive system, *NOD2* gene

## Abstract

**Background:**

Blau syndrome (BS) is a monogenic disorder caused by *NOD2* gene variants characterized by the triad of granulomatous polyarthritis, rash, and uveitis. Atypical symptoms were recognized in one-third to one-half of individuals with BS. This study aims to describe the clinical features of BS patients with hypertension and digestive system involvement.

**Methods:**

The complete clinical data of a BS patient complicated with hypertension and hepatic granulomas were collected and documented. We also performed a literature search to find all reported cases of BS with hypertension and digestive system involvement.

**Results:**

We reported the case of a 19-year-old man who presented with early onset symmetric polyarthritis and hypertension at age 5 and hepatic granulomas and cirrhosis at age 19. He was diagnosed with BS by the finding of a variant of the *NOD2* gene (R334W). Through the literature review, 24 patients with BS were found who were reported to have hypertension, and 38 patients were found who had different digestive system manifestations such as hepatic granulomas, hepatosplenomegaly, diverticulitis, and intestinal granuloma. Among the 38 BS patients with digestive system involvement, 14 had hepatic granulomas proven by liver biopsy.

**Conclusions:**

Hypertension and digestive system involvement are rare manifestations of BS. Clinicians, especially rheumatologists, must be aware of atypical symptoms of BS.

## 1. Introduction

Blau syndrome (BS) is a rare autosomal dominant inherited autoinflammatory granulomatous disorder described by Edward Blau in 1985 (1). The *NOD2* (nucleotide-binding oligomerization domain containing 2) gene has been proven to be the disease-causing gene (2, 3). The typical clinical manifestations of BS consist of granulomatous dermatitis, arthritis/periarthritis, and uveitis. More and more atypical symptoms including visceral and vascular involvements have been observed. Here, we report a rare case of BS complicated with hypertension and hepatic granulomas. We also reviewed the published English-language literature to identify all reported cases of BS with hypertension and all cases with digestive system involvement.

## 2. Materials and methods

**Figure 1:**
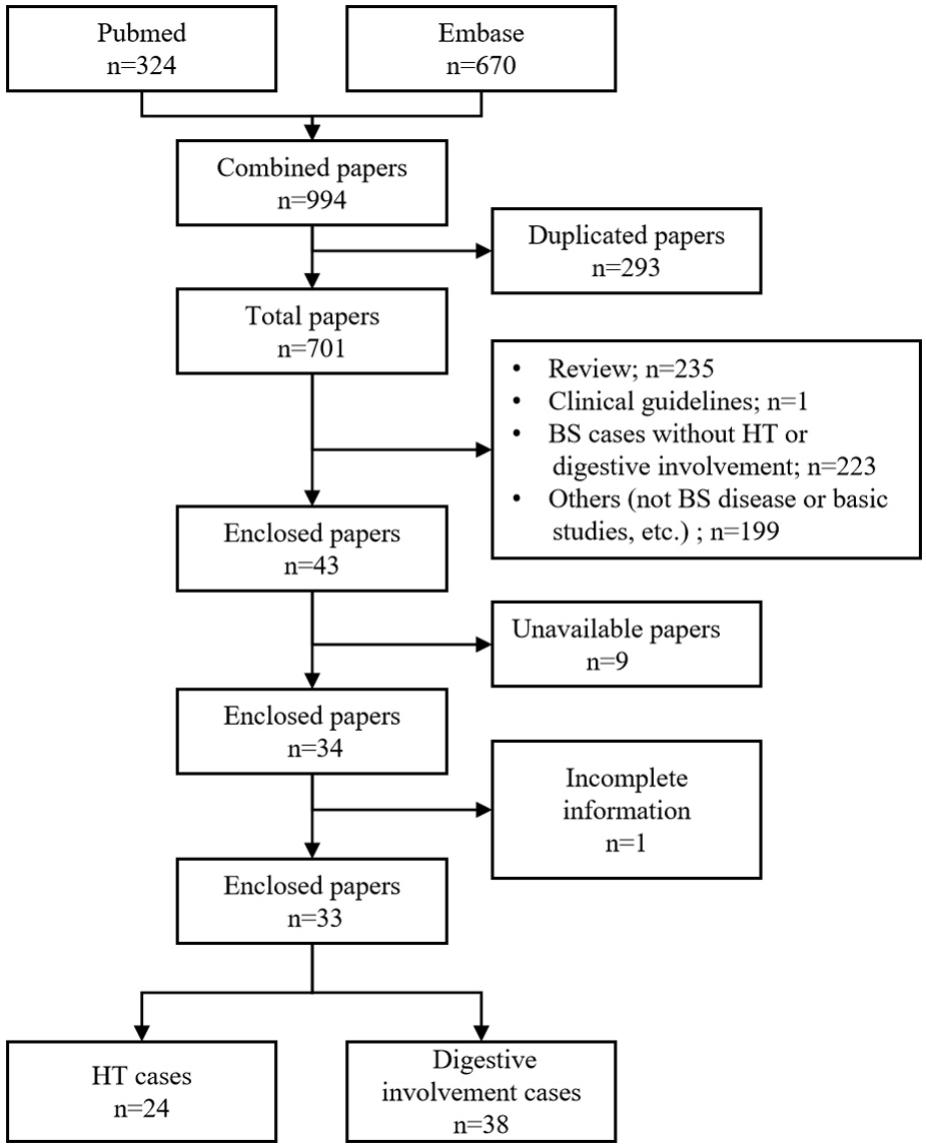
Flow chart of the literary review process (BS, Blau syndrome; HT, hypertension).

The complete medical records of this patient were collected and documented. This study was approved by the Institutional Review Board of Peking Union Medical College Hospital. Informed consent was obtained from the patient. We performed a systematic literature search in PubMed and EMBASE using the keyword “Blau syndrome” for a time period ranging from September 1991 to June 2022. Patients with BS who were found to have hypertension or digestive system involvement were included in this review. Case reports that were published in languages other than English were excluded. The review process is graphically presented in Figure 1. Finally, 33 articles containing BS cases with hypertension and/or digestive system involvement were reviewed. The vast majority of these were case reports or retrospective studies.

## 3. Results

### 3.1. Case description

**Figure 2:**
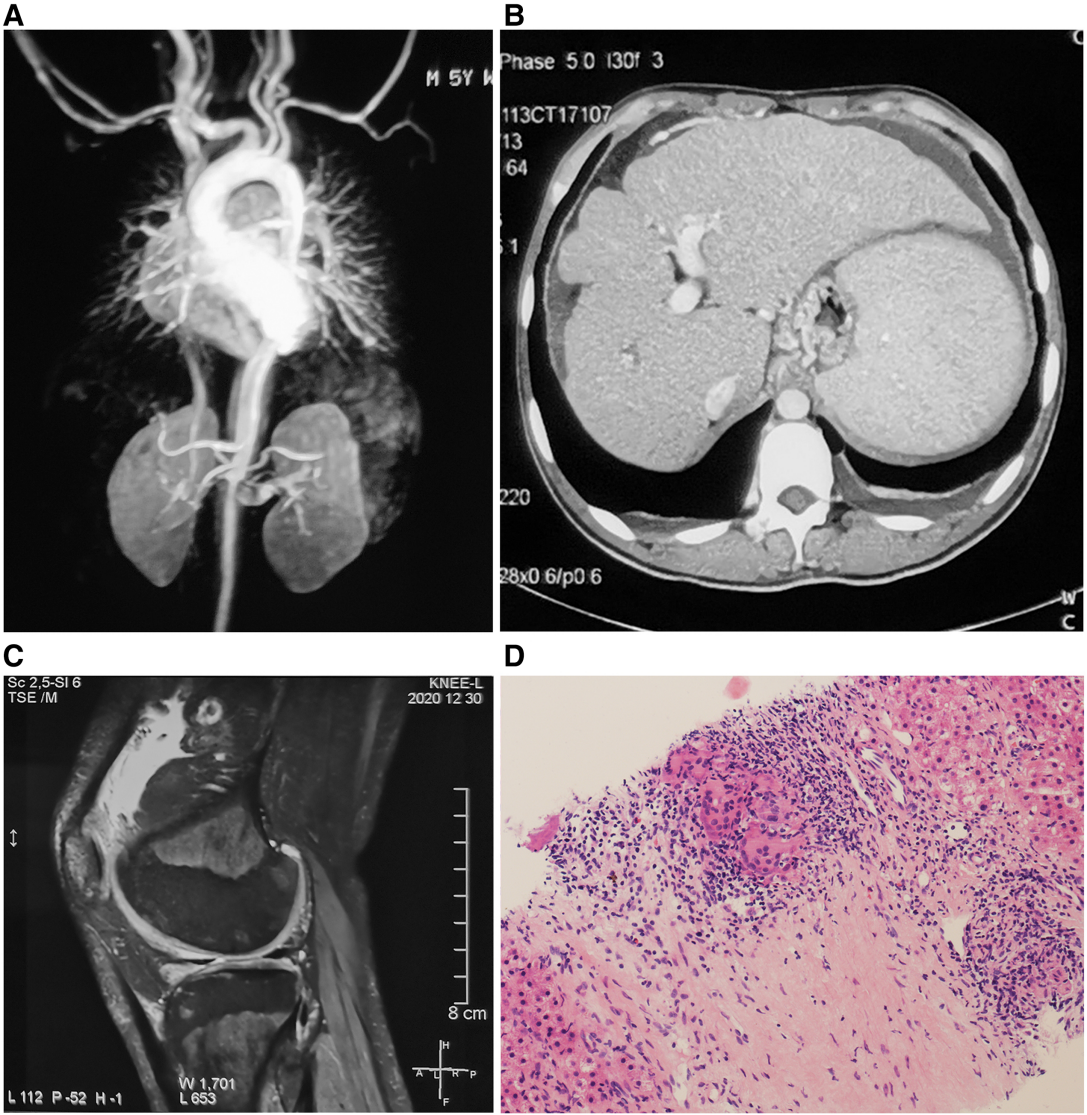
(**A**) Magnetic resonance angiography showing stenosis and thickening of the wall of the abdominal aortic. (**B**) Abdominal CT showing liver cirrhosis, splenomegaly, portal hypertension, and ascites. (**C**) Magnetic resonance imaging showing joint effusion, synovial thickening, and bone destruction of the distal femur and proximal tibia in the left knee. (**D**) Liver biopsy showing epithelioid granulomas with multinucleated giant cell reaction [hematoxylin and eosin (H&E)-stained section, 150× magnification].

A 19-year-old Chinese Han man presented with skin rash and joint contracture. Red papules had developed on his back only 1 month after birth. The rash gradually spread to his trunk and upper and lower limbs. He had no familial history of autoinflammatory diseases. At age 3, he had arthritis with symmetric periarticular swelling of wrists, knees, and ankles, and developed progressive deformity at proximal interphalangeal joints. At age 5, he was diagnosed with hypertension. In the local hospital, laboratory tests showed an erythrocyte sedimentation rate (ESR) of 27 mm/h (normal range: 0–15 mm/h), C reactive protein (CRP) of 36.5 mg/L (normal range: 0–8 mg/L), serum IgG of 18 g/L (normal range: 5.53–13.07 g/L), and IgA of 5.04 g/L (normal range: 0.33–1.08 g/L). Magnetic resonance (MR) angiography showed stenosis and thickening of the wall of the abdominal aortic at the level of the renal artery (Figure 2A) and mild hepatosplenomegaly. Skin biopsy revealed non-caseating granulomas in the superficial dermis, multinucleated giant cells, scattered lymphocytes, and infiltration of a few eosinophils. Special staining of the skin tissue, including with acid-fast staining, periodic acid-Schiff staining, and hexamine silver staining, was negative. He was still undiagnosed, and his blood pressure was well controlled with amlodipine. He was intermittently treated with diclofenac sodium for arthralgia. Eye redness had often occurred before age 10, but the cause was unknown. At age 18, he suffered from hematemesis and melena. A complete blood count found that hemoglobin (Hb) was 64 g/L (normal range: 130–175 g/L), and the platelet (PLT) count was 96 × 10^9^/L (normal range: 125–350 × 10^9^/L). The liver function test showed that serum albumin (ALB) was 32.7 g/L (normal range: 40–55 g/L), alkaline phosphatase (ALP) was 145 U/L (normal range: 45–125 U/L), γ-glutamine transferase (GGT) was 148 U/L (normal range: 10–60 U/L), total bilirubin (TBIL) was 25.56 μmol/L (normal range: 3–22 μmol/L), and direct bilirubin was 12.39 μmol/L (DBIL) (normal range: 0–8 μmol/L). Hepatitis serologies for hepatitis B and C, ceruloplasmin, and autoimmune liver disease-related autoantibodies were negative. Abdominal computed tomography (CT) showed liver cirrhosis, splenomegaly, portal hypertension, and ascites (Figure 2B). Gastroscopy revealed esophageal and gastric varicose veins (F3 varices, gastroesophageal varices type 1) and portal hypertensive gastropathy. MR imaging showed joint effusion with extensive synovial thickening and bone destruction of the distal femur and proximal tibia in the left knee (Figure 2C). A liver biopsy revealed cirrhosis and hepatic granulomas (Figure 2D). He was given red-blood-cell transfusion and endoscopic injection sclerotherapy for esophageal varices and was transferred to our hospital.

**Figure 3:**
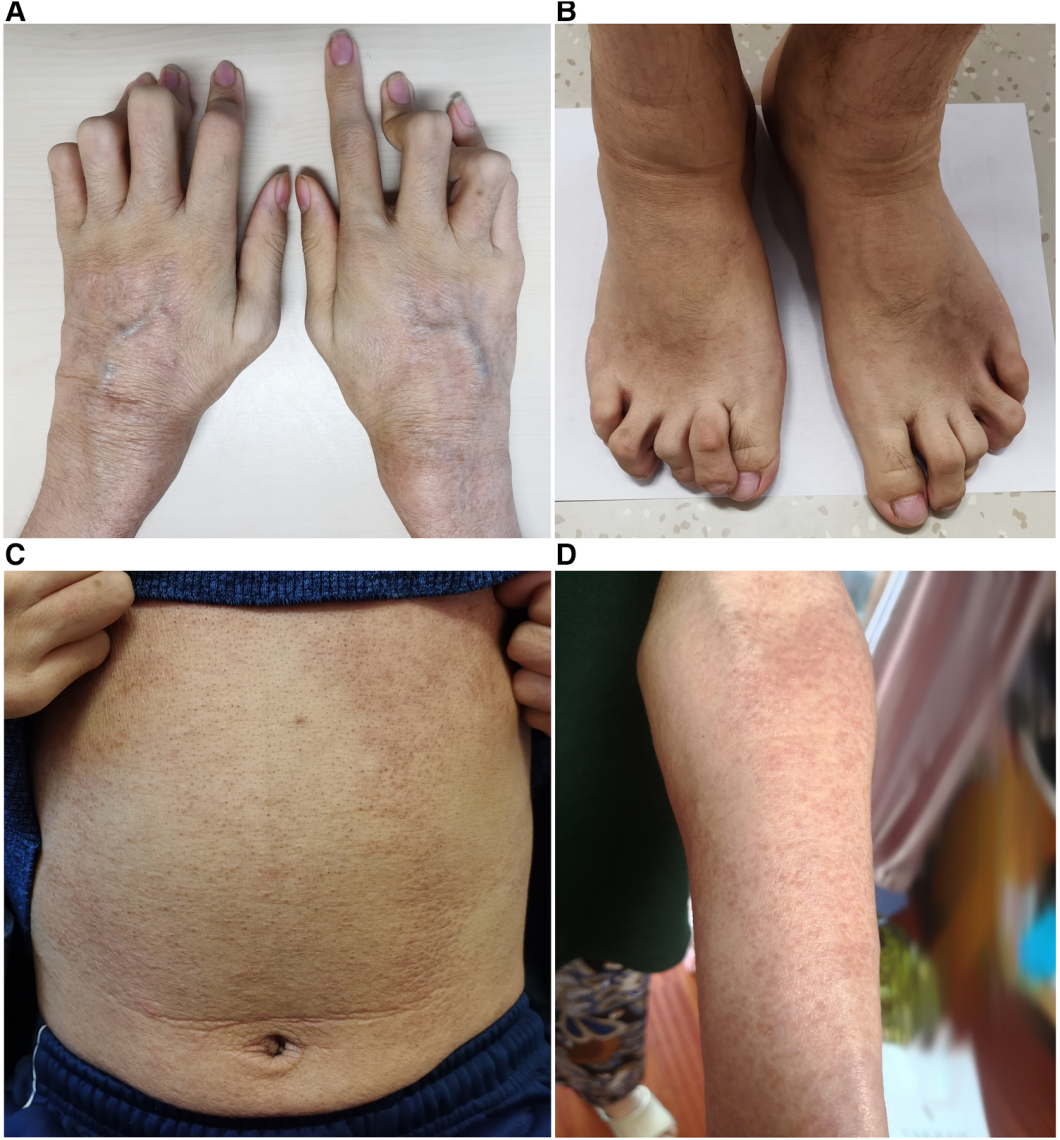
(**A,B**) Camptodactyly of the fingers and toes. (**C,D**) Ichthyosiform rashes on the trunk and limbs.

On physical examination, he appeared with camptodactyly (Figures 3A,B) and boggy swelling of both wrists and knees. Ichthyosiform rashes on the whole body (Figures 3C,D) and splenomegaly were noted. Further whole exome sequencing by Next-Generation Sequencing identified a *de novo* pathogenic heterozygous variant of the *NOD2* gene (NM_022162, Exon4), c.1000C>T, p.R334W. He was diagnosed with BS and treated with subcutaneous adalimumab, 40 mg once every 2 weeks. The rash and joint symptoms improved, but he had repeated gastrointestinal bleeding. He received a transjugular intrahepatic portosystemic shunt (TIPS) combined with balloon-occluded retrograde transvenous obliteration thereafter. At 1-year follow-up, the rash and arthritis had relapsed. Adalimumab was discontinued, and canakinumab was given subcutaneously 150 mg every 8 weeks. At the last follow-up, his symptoms had become stable. Laboratory tests showed the following results: Hb: 82 g/L, WBC: 2.01 × 10^9^/L, PLT: 63 × 10^9^/L, ALB: 30.9 g/L, GGT: 75 U/L, ALP: 186 U/L, TBIL: 50.63 μmol/L, DBIL: 30.88 μmol/L, IgG: 21.9 g/L, and PT: 14.1 s. Blood ammonia, CRP, and ESR were all normal.

### 3.2. Systematic literature review

Among the 33 English language articles we found of patients with BS, there were 24 cases with hypertension (4–19) and 38 cases with digestive system involvement (5–7, 11–13, 18–36) (Supplementary Tables S1, S2). The 24 patients with hypertension included 10 men, nine women, and five with unknown gender, with a median age at baseline evaluation of 17.1 years (range: 4–57). The 38 patients with digestive system involvement comprised 15 men, 18 women, and five with unknown gender, with a median age at baseline evaluation of 20.3 years (range: 1.4–57). Of the 38 patients with digestive system involvement, 14 had hepatic granulomas proven by liver biopsy (5, 7, 11, 16, 20–27); five patients with hepatic granulomas had liver cirrhosis (21, 23, 24, 26, 27); 24 had enlarged liver and/or spleen (5, 11, 12, 15, 16, 18, 22, 26, 27, 29–35); six had elevated liver enzymes including transaminase and cholestatic liver enzyme (21–24, 26, 27); five had gastrointestinal diseases including gastric ulcer, diverticulitis, Barrett's esophagus and small and large intestine granulomas (6, 13, 36); one had multiple splenic and hepatic lesions (28); and one had fatty liver (13) (Supplementary Table S2).

## 4. Discussion

BS is an autosomal dominantly inherited disorder caused by gain-of-function variants in the caspase recruitment domain of *NOD2*. *NOD2* recognizes MDP. Dysfunctional *NOD2* is overactive in that context (3, 37, 38). For most patients with BS, the disease is characterized by early onset, typically at ages before 3–4 years (39). Articular and cutaneous symptoms are often the initial manifestations (5, 31, 40), followed by ocular symptoms several years later. Joint manifestations usually appear as symmetric polyarthritis, periarticular swelling, and tenosynovial cysts due to frequent granulomatous inflammation in the periarticular structures (5, 40). Articular deformities with camptodactyly are typical in BS, causing moderate-to-severe joint dysfunction. The rash often presents as erythema with maculopapular configuration located on the trunk and/or limbs (39). Granulomatous uveitis is a typical feature of BS and can lead to blindness. Cataract, chorioretinitis, and glaucoma can also be observed in more than one-third of patients (41, 42). Not all patients have the typical triad (43). Our patient only had articular and cutaneous symptoms without ocular lesions.

One-third to one-half of individuals with BS present atypical symptoms including vascular and visceral involvements (7, 11, 29). Vascular involvement includes hypertension, large-vessel vasculitis, ischaemic stroke, leukocytoclastic vasculitis, and pulmonary embolism (6, 7, 9–11, 14, 15, 44–49). Visceral involvement includes interstitial nephritis, hepatic granulomas, renal granulomas, hepatomegaly, splenomegaly, generalized lymphadenopathy, interstitial lung disease, sensorineural hearing loss, pericarditis, and so on (5, 7, 11, 15, 23, 26, 29, 49, 50). Recurrent fever also frequently develops in patients with BS (15).

Systemic hypertension is rare in BS. Patients can develop hypertension during the early phases of the disease. In our literature review, two patients even became hypertensive before age 5 (10, 12). The underlying mechanism is probably multifactorial. In our study, seven patients presented with large-vessel vasculitis resembling Takayasu arteritis, just like our patient. Of the seven cases, one had only renal artery stenosis (14), five had aortic stenosis other than renal artery stenosis (9, 10, 15, 17, 18), and in one, other artery involvement was not specified, except renal artery stenosis (11). Ten patients were reported to have other forms of renal diseases, such as chronic renal failure, granulomatous nephritis, tubulointerstitial nephritis, renal tubular dysfunction, and nephrocalcinosis (4–8, 11, 12, 16, 19). One patient was reported with renal clear cell carcinoma (8). The hypertension of the aforementioned patients may be attributable to renal hypertension secondary to BS. It has also been reported that BS patients developed hypertension secondary to long-term use of corticosteroids (11). Still, some BS patients with hypertension did not have the above reasons. Intriguingly, one patient even developed hypertension 21 years earlier than renal artery stenosis (14). The mechanism of hypertension in BS is unclear, and vasculitis of large arteries is an important cause, as we described above. However, the pathogenesis of large-vessel vasculitis in BS has not been elucidated. Every patient with hypertension should be evaluated for vasculitis, and blood pressure should be monitored closely. With regard to the treatment of hypertension, some patients used angiotensin-converting enzyme inhibitors with dramatically positive responses. So, it is presumed that these patients had renal hypertension. However, some patients needed a combination of multiple antihypertensive drugs to control hypertension. There were no reports that blood pressure could be controlled after immunosuppressive therapy without antihypertensive drugs.

Digestive system involvement is also rare in BS and includes gastrointestinal, liver, and spleen involvements, yet pancreatic or gallbladder involvements have not been described. Digestive system involvement in BS patients commonly manifested as abdominal pain, diarrhea, ascites, jaundice, hematemesis, and melena (27, 31, 35), although many patients were asymptomatic and their gastrointestinal involvement was discovered during the medical evaluations. In our literature review, gastrointestinal involvement confirmed by gastroenteroscopy included gastric ulcer, diverticulitis, and Barrett's esophagus. However, with such a small number of cases, it is difficult to draw firm conclusions regarding a clear relationship between the above non-granulomatous gastrointestinal diseases and BS. Interestingly, small and large intestine granulomas were reported in a single case (6). Spleen involvement mainly manifested as splenomegaly by physical examination or imaging. Liver involvement was the most frequent among digestive system organs involved in BS. Thirty-one patients were reported with liver involvement, which mainly manifested as elevated liver enzymes, hepatomegaly, and hepatic granulomas. One patient had fatty liver, but this might have been a coincidental occurrence since he was already 57 years old (13). There were 14 patients with hepatic granulomas (5, 7, 11, 16, 20–27). It has been reported that patients with BS may develop granulomas at unusual locations, such as the liver, intestines, parotid glands, and kidneys (26). The incidence of liver granuloma is slightly higher than other unusual locations (26). Of the 14 patients we reviewed, it was found that five patients were carrying heterozygous p.R334Q *NOD2* mutations, two were carrying heterozygous p.C495Y mutations, two were carrying heterozygous p.E383D mutations, two were carrying heterozygous p.M513T mutations, and the remaining three patients were carrying a variety of different *NOD2* mutations (E498G, E268S, and M491l). These 14 patients comprised four men, eight women, and two with unknown gender. Hepatic granuloma was diagnosed in an age range from 17 months to 48 years. In addition to the typical triad, hepatic granuloma in patients may also be complicated by granulomas of other unusual locations and other atypical manifestations such as erythema nodosum. Liver histology mostly showed non-caseating epithelioid granulomas containing lymphocytes, macrophages, epithelioid cells, and multinucleated giant cells, which suggests an abundant inflammatory response. Unfortunately, five patients with hepatic granulomas were diagnosed with liver cirrhosis simultaneously (21, 23, 24, 26, 27), resulting in portal hypertension, esophageal varices, jaundice, and ascites. Patients with cirrhosis (median age 24.7 years old) were generally older than those without cirrhosis (median age 14.8 years old), suggesting that patients with liver granuloma may develop cirrhosis in the future, and it is a potentially serious consequence of the disease.

The specific treatment for BS, especially with atypical symptoms, has not been fully established. Because of the rarity, there is also a lack of research on the treatment effect of large BS cohorts. Glucocorticoids, immunosuppressants (such as methotrexate and azathioprine), and biologics including TNF inhibitors, tocilizumab, tofacitinib, and baricitinib have good therapeutic effects and may improve the prognosis of BS (27, 35, 36, 51). The joint and skin symptoms in our patient improved after adalimumab treatment for about 1 year, but his hepatic disease showed no response to either adalimumab or canakinumab. In our literature review, few follow-up data are available on hepatic disease in BS patients. Some patients developed hepatic granulomas and even liver cirrhosis during the use of glucocorticoids or immunosuppressants, and one patient even developed hepatic granulomas after treatment with etanercept (20). It was reported that liver enzymes in two patients with hepatic granulomas decreased after adalimumab treatment (21, 26), and MR elastography improved liver stiffness in one of them (21). Hepatosplenomegaly in one patient was resolved by infliximab combined with glucocorticoids (11). Hepatomegaly, but not splenomegaly, in one patient was resolved by glucocorticoids (11). In another article, no response to methotrexate and glucocorticoid treatment was observed, with persistence of hepatomegaly. Moreover, there is also a case report of progressive cirrhosis after adalimumab treatment. That patient eventually had two liver transplants, and a biopsy of the first transplanted liver showed disease recurrence (27). Therefore, preventing the progression of liver manifestations in BS patients remains challenging, and large-scale studies and long-term follow-up are needed.

## 5. Conclusions

BS is a childhood-onset autoinflammatory disease manifesting as a clinical triad of granulomatous dermatitis, arthritis, and recurrent uveitis. Hypertension and digestive system involvement are rare complications of BS. The mechanisms of hypertension and non-granulomas gastrointestinal involvement in BS are still unclear. Clinicians, especially rheumatologists and pediatric rheumatologists, must be aware of its clinical manifestations. Early diagnosis and prompt treatment may contribute to preventing the occurrence of serious complications.

## Data Availability

The original contributions presented in the study are included in the article/**Supplementary Material**; further inquiries can be directed to the corresponding authors.
